# The mechanistic role of natural antimicrobials in preventing *Staphylococcus aureus* invasion of MAC-T cells using an in vitro mastitis model

**DOI:** 10.1186/s13620-024-00265-0

**Published:** 2024-02-27

**Authors:** Igori Balta, David McCleery, Saida Roxana Feier David, Elena Pet, Ducu Stef, Tiberiu Iancu, Ioan Pet, Lavinia Stef, Nicolae Corcionivoschi

**Affiliations:** 1 Faculty of Bioengineering of Animal Resources, University of Life Sciences King Mihai I from Timisoara, Timisoara, 300645 Romania; 2 Bacteriology Branch, Veterinary Sciences Division, Agri-Food and Biosciences Institute, Northern Ireland, Belfast, BT4 3SD UK; 3 Faculty of Management and Rural Development, University of Life Sciences King Mihai I from Timisoara, Timisoara, 300645 Romania; 4 Faculty of Food Engineering, University of Life Sciences King Mihai I from Timisoara, Timisoara, 300645 Romania

**Keywords:** *Staphylococcus aureus*, Mastitis, Natural antimicrobials, Bovine mammary epithelial cells, ClfB, AnxA2

## Abstract

**Background:**

Starting primarily as an inflammation of the mammary gland, mastitis is frequently driven by infectious agents such as *Staphylococcus aureus*. Mastitis has a large economic impact globally, which includes diagnostic, treatment, and the production costs not to mention the potential milk contamination with antimicrobial residues. Currently, mastitis prevention and cure depends on intramammary infusion of antimicrobials, yet, their overuse risks engendering resistant pathogens, posing further threats to livestock.

**Methods:**

In our study we aimed to investigate, in vitro, using bovine mammary epithelial cells (MAC-T), the efficacy of the AuraShield an antimicrobial mixture (As) in preventing *S. aureus* attachment, internalisation, and inflammation. The antimicrobial mixture (As) included: 5% maltodextrin, 1% sodium chloride, 42% citric acid, 18% sodium citrate, 10% silica, 12% malic acid, 9% citrus extract and 3% olive extract (w/w).

**Results and discussion:**

Herein we show that As can significantly reduce both adherence and invasion of MAC-T cells by *S. aureus*, with no impact on cell viability at all concentrations tested (0.1, 0.2, 0.5, 1%) compared with untreated controls. The anti-apoptotic effect of As was achieved by significantly reducing cellular caspase 1, 3 and 8 activities in the infected MAC-T cells. All As concentrations were proven to be subinhibitory, suggesting that Ac can reduce *S. aureus* virulence without bacterial killing and that the effect could be dual including a host modulation effect. In this context, we show that As can reduce the expression of *S. aureus* clumping factor (ClfB) and block its interaction with the host Annexin A2 (AnxA2), resulting in decreased bacterial adherence in infection of MAC-T cells. Moreover, the ability of As to block AnxA2 had a significant decreasing effect on the levels of pro inflammatory cytokine released upon *S. aureus* interaction with MAC-T cells.

**Conclusion:**

The results presented in this study indicate that mixtures of natural antimicrobials could potentially be considered an efficient alternative to antibiotics in treating *S. aureus* induced mastitis.

## Background

Maintaining optimal udder health in dairy farming requires a strong emphasis on hygiene practices, with the disinfection of teats emerging as a predominant preventive measure against mastitis, method commonly referred to as “post-dipping” [1]. Bovine mastitis (BM) continues to challenge the dairy sector as it’s a leading communicable health condition, causing substantial economic consequences for farmers worldwide [2]. Commercial disinfectants typically incorporate a range of antimicrobial agents, such as chlorhexidine, iodine, hydrogen peroxide, lactic acid, sodium hypochlorite, and triclosan. However, the widespread use of these chemicals poses several concerns. Not only do they have the potential to compromise the integrity of dairy products by leaving residues in milk, but they can also introduce environmental contaminants [1]. Microorganisms such as *Staphylococcus aureus*, *Escherichia coli, Streptococcus dysgalactiae*, *Streptococcus agalactiae*, *Streptococcus uberis, Pseudomonas aeruginosa* and *Klebsiella* are notorious for their prevalence in BM which collectively complicate therapeutic strategies [3–5]. Out of the approximately 250 infectious agents implicated in bovine mastitis, *Staphylococcus* spp., in particular, is the predominant causative agent, linked with a myriad of symptoms [2].

Alarmingly, a significant fraction of *Staphylococcus* spp. derived from mastitis cases have been reported to display antimicrobial resistance. The notorious pathogen, *S. aureus*, has earned its reputation for its persistent infections with low recovery rates, largely due to its ability to form biofilms, evade host immune responses, and reside within micro-abscesses [2]. Recent trends have also witnessed a surge in mastitis cases associated with coagulase-negative *Staphylococci* (CNS), capable of producing toxins and interacting with fibronectin-binding proteins (FnbA) [2, 6]. *FnbA* and *ClfA* are adhesive proteins facilitating the pathogen’s engagement with bovine mammary epithelial cells, playing a pivotal role in biofilm genesis [7]. Concurrently, *SigB* is instrumental in modulating stress-responsive and survival genes. It also steers biofilm development via the *SigB* regulon, a crucial factor for perpetuating chronic infections [7]. Additionally, *ArlR* is an integral part of the *ArlRS* signaling pathway, influencing both virulence determinants and antibiotic resistance by directly modulating subsequent gene expression [7]. The transmission of *staphylococci* frequently occurs between animals and humans with both populations exhibiting a significant presence of *staphylococcus* spp [6].

Bovine mastitis’s etiology can be attributed to an array of factors, ranging from contagious pathogens, nutritional inadequacies to inadequate farm management practices. Clinically, this disease is marked by conspicuous milk alterations like elevated coagulant levels, often accompanied by other prominent symptoms in the animal, including fever, clear signs of infection and inflammation, discernible upon inspection [4]. In contrast, subclinical mastitis (SCM) poses diagnostic challenges; cows typically seem unaffected and neither the udder nor the milk displays noticeable changes [8]. A particular subset of bovine mastitis induced by environmental pathogens exhibits a spike in the somatic cell count (SCC), which carries its own economic burdens owing to the enhanced cell counts in milk [8]. Notably, SCM, despite its covert nature, is the more common form and incurs greater economic losses than its clinical counterpart [4]. However, the implications of *Staphylococci* extend beyond the realm of bovine health as they are prominent nosocomial pathogens, with over 50 *Staphylococcus* spp and subspecies being implicated in bovine staphylococcal mastitis [6]. The transmission dynamics of resistance genes and direct zoonotic transmission raise public health concerns [9]. Additionally, some strains of *S. aureus* are recognized as potent foodborne pathogens, causing food poisoning [10]. The interplay between human and animal environments further elevates the risks, given the high prevalence and propensity of *Staphylococci* to transition between these domains.

Given the increasing antibiotic resistance exhibited by *S. aureus* and the formidable challenges posed by its biofilm production, recent scientific endeavors are veering towards more novel, natural remedies. There is an escalating trend towards harnessing the antimicrobial properties of certain natural antimicrobials, herbal substances, peptides and probiotics that exhibit potent activity against a spectrum of pathogens, including not just *S. aureus*, but also the likes of *Campylobacter* spp., *Salmonella* spp., *E. coli*, and *Clostridium* spp among others [5, 6, 10–15]. The aim of the current study was to bring further clarity regarding the mechanisms by which natural antimicrobials can reduce *S. aureus* infection of bovine mammary cells. More specifically, we have examined the molecular mechanisms by which natural antimicrobials can block the interaction between the pathogen and host.

## Methods

### Microbiology and cell culture and antimicrobial mixture

*Staphylococcus aureus* (S. aureus DSM1104)—clinical isolate (laboratory stock), was grown in TSAYE (Tryptone Soya Yeast Extract) at 37 °C. Cultures were suspended in phosphate buffered saline (PBS; Gibco, UK) to an optical density (OD_600_) of 1 prior to dilution in appropriate culture medium (FLUOstar Omega, BMG Labtech, U.K.). Colony forming units were enumerated by manual count following serial dilution in PBS, spreading onto tryptic soy agar and overnight incubation at 37 °C. MAC-T cells, a bovine mammary epithelial cell line (ATCC CRL-10,274) was cultured in Dulbeccos’ Modifed Eagle medium (DMEM; Sigma, UK) with 10% foetal bovine serum (FBS; Sigma) at 37 °C, 5% CO_2_. AuraShield (As) included: 5% maltodextrin, 1% sodium chloride, 42% citric acid, 18% sodium citrate, 10% silica, 12% malic acid, 9% citrus extract and 3% olive extract (w/w). Bioscience Nutrition, Fedamore, Ireland supplied the raw materials.

### Growth curves

*S. aureus* cultures (2.0 log CFU/ml in TSAYE) were transferred into 96 well microtiter plates with final concentrations of the natural antimicrobial of 0, 0.1, 0.2, 0.5, 1 and 2% As (v/v). The inoculum level was determined after plating out onto TSAYE plates and incubation at 37 °C for 24 h. Cultures were incubated at 37 °C, and optical density was monitored at 600 nm at intervals of 4 h, over 20 h, using an automatic plate reader (FLUOstar Omega, BMG Labtech, U.K.). The concentrations of the antimicrobial that did not inhibit bacterial growth were chosen as the sub-inhibitory concentrations and used for the subsequent phenotypic virulence assays.

### Cell infections

Firstly, for *S. aureus* infection studies, the MAC-T cells were seeded in 6-well plates at 10^4^ cells/well in 2 ml DMEM containing 10% FBS. Cells were then incubated at 37 °C in 5% CO_2_ until 80% confluent. Cells were washed (2X) with pre-warmed DMEM containing 1% FBS before inoculation with *S. aureus* at a multiplicity of infection (MOI) of 10. As controls uninoculated wells were used. Cells were infected for 6 h of incubation at 37 °C in 5% CO_2_ in the presence of 0, 0.1, 0.2, 0.5% and 1% AuraShield (As) for 2–3 h. Post infection the supernatant, containing un-attached bacteria was removed and wells were washed with 1 ml pre-warmed PBS. The cytotoxicity of As was determined as previously described [13] using the MTT assay (Sigma-Aldrich, Gillingham, England, UK). To quantify the number of cell-associated bacteria, infected monolayers were washed three times with PBS and treated with 0.1% Triton X-100 in PBS at 41.5 °C and 37 °C for 15 min. Ten-fold dilutions from each well were plated onto TSAYE agar and the colonies were enumerated after 2 days of incubation at 37 °C. The infected monolayers were washed with tissue culture medium to quantify the number of bacteria that invaded MAC-T cells. Fresh medium (2 ml) containing gentamicin (400 µg/ml) was added to kill bacteria that were not internalized. Medium without gentamicin was introduced to quantify the number of bacteria that adhered to the epithelial cells. Next, the tissue culture plates were incubated for a further 3 h at 41.5 °C or 37 °C and washed with fresh DMEM + 10% FBS. MAC-T cells were lysed by the addition of 1 ml of 0.1% Triton X-100 in PBS and incubated for 15 min at 41.5 °C or 37 °C. Tenfold dilution of each well content was plated onto TSAYE agar, and colonies were enumerated after 1–2 days of incubation. Invasion efficiency was calculated as the percentage of the total number of CFU/total initial inoculum. Secondly, we have set up infections to investigate the impact on ClfB expression. In this case the bacteria grown in the presence of 0, 0.1, 0.2, 0.5 and 1% As, the bacteria present in the supernatant of the infected cells and the bacteria attached and internalised were investigated. Thirdly, to study the implication of Annexin A2 (AnxA2) we have pre-incubated MAC-T cells with anti-AnxA2 antibody (17.5 µg/ml) or human IgG (17.5 µg/ml) (Themo Fisher Scientific, UK) for 3 h prior to infection to block AnxA2 function as previously described [16]. For this latest experiment only the 1% As concentration was used, versus 0% as a control. All assays were conducted in triplicate on three separate days. The significance of differences in adhesion and invasion between samples was determined using the Student’s *t*-test. A *P*-value of < 0.05 was defined as significant (*n* = 3).

### Gene expression analysis

The quantification of IL6, IL-1β, IL-8 and TNFα gene expression was conducted as previously described [17]. Briefly, the infected MAC-T cells were frozen in liquid nitrogen until use. The RNeasy Plus Mini Kit (Qiagen, Manchester, UK) kit was used for RNA isolation. Reverse-transcribed RNA was obtained by using the Transcriptor First Strand cDNA Synthesis Kit (Roche, Dublin, Ireland). The primers used. cDNA (1 µl) was added to duplicate wells with 7 µl of DNase-free water, 10 µl of Taqman Fast Advanced master mix (Applied Biosystems) and 1 µl of each of 2 Taqman gene expression assays (Applied Biosystems) (*IL-6* NM_173923.2 Bt03211903_m1 FAM 69, *IL-8* NM_173925.2 Bt03211907_g1 FAM 105, *TNFα* NM_173966.3 Bt03259155_g1 VIC 66, *IL-1β* NM_174093.1 Bt03212744_m1 FAM 69). Samples were incubated at 50 °C for 2 min followed by 95 °C for 20 s then cycled 40 times at 95 °C for 3 s and 60 °C for 30 s in a LightCycler 96 (Roche). ClfB gene expression was analysed as previously described [18]. The extraction of RNA was performed with chloroform and purified using the RNeasy minikit (Qiagen, UK). Fold change in gene expression level was calculated by comparing to Cq values in the absence of the natural antimicrobials. The purified RNA was reverse transcribed into cDNA by using Transcriptor First Strand cDNA Synthesis Kit (Roche, Dublin, Ireland) according to the manufacturer’s protocol. TaqMan primers (F-aatgtgttaccactttgattagggtcaa, R-gctgctgatgctaaaggtacaaatg and probe-acggcaagtaatttc containing a 6-carboxyfluorescein reporter and nonfluorescent quencher were designed using the TaqMan design tool (Life Technologies). cDNA samples were assayed in triplicate using 16 S rRNA as a control. The 2^–ΔΔCT^ method was used to analyse the relative expression (fold changes), calculated relative to the control group. The expression of GAPDH gene was used as a control for MAC-T internalised *S. aureus*.

### H_2_O_2_ production in infected MAC-T cells

The production of intracellular ROS was measured using 2′,7′-dichlorofluorescein diacetate (DCFH-DA) as previously described [19]. Briefly, a 10 mM DCFH-DA stock solution (in methanol) was diluted 500-fold in PBS to obtain a 20 µM working solution. After infection, with or without As, the cells in a 12-well plate were washed twice with PBS and then incubated in a 100-µL working solution of DCFH-DA at 37 °C for 30 min. NADPH inhibitors including diphenyleneiodonium chloride (DPI, Sigma; 15 µM, 45 min preincubation and wash out) and bovine liver catalase (Sigma-Aldrich, Gillingham, England, UK; 300 U/ml) were used during the 6 h measuring interval. Fluorescence was then determined with an excitation wavelength of 485 nm and an emission wavelength of 520 nm using a microplate reader (FLUOstar Omega from Premier Scientific, Belfast, UK). The ROS levels are expressed as fold change over the infected and treated control.

### Caspase activity

Caspase 1, 8, and 3 enzymatic activities were measured in infected MAC-T cells as previously [20]. Cells were infected for 6 h, in the presence of 0, 0.1, 0.2, 0.5 and 1% As described above. Briefly, the infected monolayers were washed 3X with sterile phosphate-buffered saline (PBS at pH 7.2), scraped of the flask surface and collected by centrifugation at 450 ×g for 5 min at 4 °C. The resulting pellets were washed again with sterile PBS and resuspended in 10 ml of clean sterile PBS. Following enumeration with a hemacytometer cells were resuspended at a concentration of 10^8^ cells/ml in cell lysis buffer (25 mM HEPES [pH 7.5], 5 mM MgCl2, 5 mM EDTA, 5 mM dithiothreitol, 2 mM phenylmethylsulfonyl fluoride, 10 µg of leupeptin per ml; all reagents from Thermo-Fisher Scientific, UK) centrifuged and the supernatants were retained. Caspase 1 activity was measured using the Caspase-Glo 1 (Promega, UK), Caspase 3 using the EnzChek kit (Thermo-Fisher Scientific, UK) and Caspase 8 was measured using the CaspGlow kit (Thermo-Fisher Scientific, UK) according to the manufacturer’s recommendations.

## Results

### *S. aureus *infection of MAC-T cells is reduced by the antimicrobial mixture (As)

**Fig. 1:**
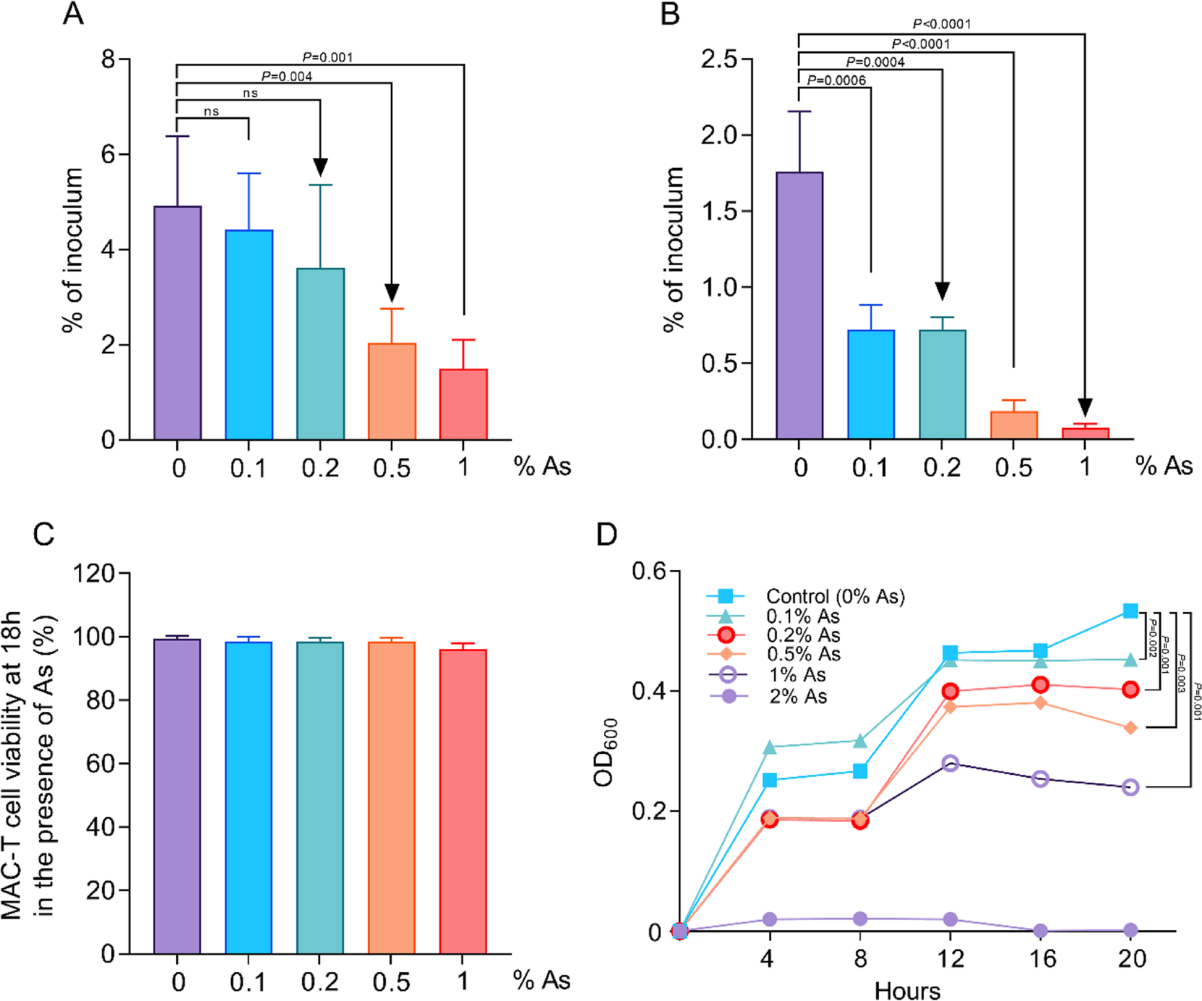
Impact of As on *S. aureus* adhesion (**A**) and infection (**B**) of MAC-T cells. The MAC-T cell viability after exposure to different concentrations of As by MTT assay is presented in panels (**C**) and the *S. aureus* growth curves are presented in panel **D**. Cell viability is expressed as a percentage of control cells (assigned as 100%). All experiments were performed in triplicate and the results are represented as means ± standard deviation (SD). Student’s *t* test was performed to assess significance with the *P* values being indicated on graphs

Bovine mammary gland epithelial cells (MAC-T cells) were used to test the effect of AuraShield concentrations (0, 0.1, 0.2, 0.5 and 1% As) on to ability of *S. aureus* to cause infection. In our study we have looked at the impact on total bacterial adhesion and on the numbers of bacteria which have penetrated the cell membrane and internalised into MAC-T cells. Our data shows a decreasing trend on the total bacterial adhesion in the presence of 0.1 and 0.2% As (Fig. 1A), however, adhesion was reduced (*P* < 0.05) at 0.5 and 1% As (Fig. 1A). The impact on bacterial internalization was nonetheless significant at all concentrations (Fig. 1B). All the above results were achieved with no impact on cell viability (Fig. 1C), suggesting that As impacts bacterial growth and their virulence mechanisms to diminished infective ability. To test this hypothesis, we have performed growth curves at all the above concentrations and observed a slower growth rate but with no growth inhibition at all the concentrations tested (Fig. 1D). This result indicated that concentrations between 0.1 and 1% As can be further used to investigate the mechanisms involved in reduced *S. aureus* infection of MAC-T cells.

### AuraShield blocks *S. aureus *ClfB expression and reduces the release of pro-inflammatory cytokines in infected MAC-T cells

**Fig. 2:**
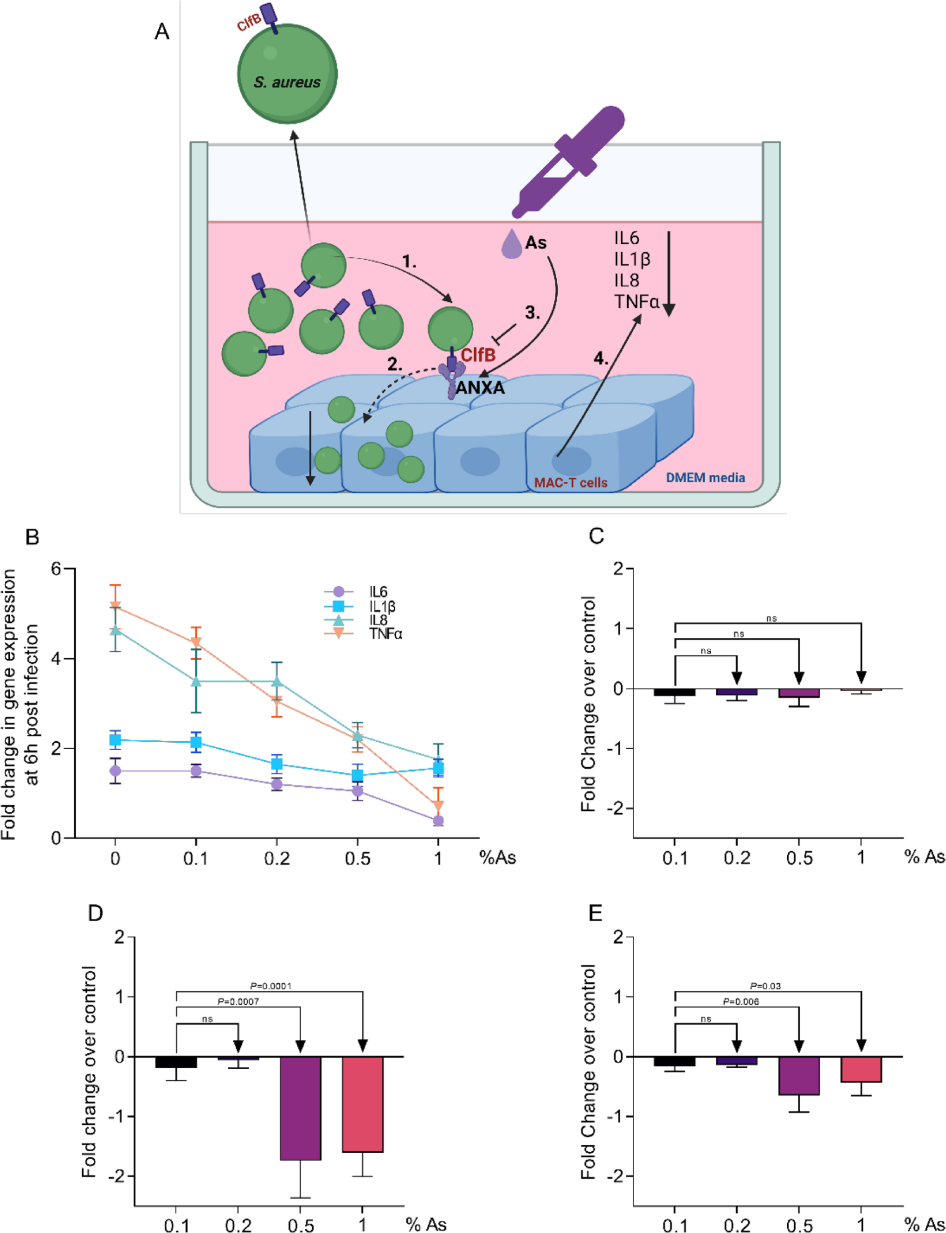
The impact of As on *S. aureus* ClfB expression and MAC-T cells inflammatory response. **A** describes the experimental design (1-exposure of *S. aureus* to MAC-T cells; 2-adhesion/infection of MAC-T cells with *S. aureus* to; 3-As inclusion during infection and inhibition of ClfB; 4-MAC-T cells pro-inflammatory response). **B** shows the impact of As on IL6, IL1β, IL8 and TNFα expression in infected MAC-T cells. **C** indicates the effect of As on ClfB expression in *S. aureus* without the presence of MAC-T cells; the expression ClfB in *S-aureus* co-cocultured with MAC-T cells and As is presented in panel **D**, **E** indicates the expression of ClfB in *S. aureus* MAC-T cells attached or internalised after 6 h of exposure to 0.1, 0.2, 0.5 and 1% As. All experiments were performed in triplicate and the results are represented as means ± SD. Student’s *t* test was performed to assess significance with the *P* values being indicated on graphs. The ClfB gene expression is presented as fold change over the infected and As unexposed control. Panel A was created using Biorender

With this experiment we aimed to gain an insight into the mechanism involved in anti-inflammatory and inflection blocking role of As during *S. aureus* infection of MAC-T cells (Fig. 2A). Our first observation indicated that the presence As during the 6 h infection of S. *aureus* infection of MAC-T cells led to a dose dependent and significant reduction in IL6, IL1β, IL8 and TNFα produced by the infected cells (Fig. 2B). To further deepen our investigation, we have assessed the impact on the *S. aureus* gene, ClfB, a clamping factor involved in adhesion. Our results show that in the absence of MAC-T cells there was no significant difference in *S. aureus* ClfB expression (Fig. 2C) indicating that the presence of MAC-T cells acts as a trigger that stimulates ClfB expression. The ClfB expression in the un-attached *S. aureus* to MAC-T cells upon infection started to increase and was significantly downregulated especially at 0.5 and 1% As (Fig. 2D). The vital role of As was further emphasized when the ClfB expression was analysed in attached and infected of MAC-T cells (Fig. 2E). The downregulation was significant at As concentration investigated. Taken together our results showed that the presence of As during MAC-T cell infection of *S. aureus* led to reduced proinflammatory cytokine release and has an expression blocking effect against the *S. aureus* ClfB clamping factor gene.

### AuraShield inactivation of AnxA2 contributes to the reduction in *S. aureus *internalization in MAC-T cells

**Fig. 3:**
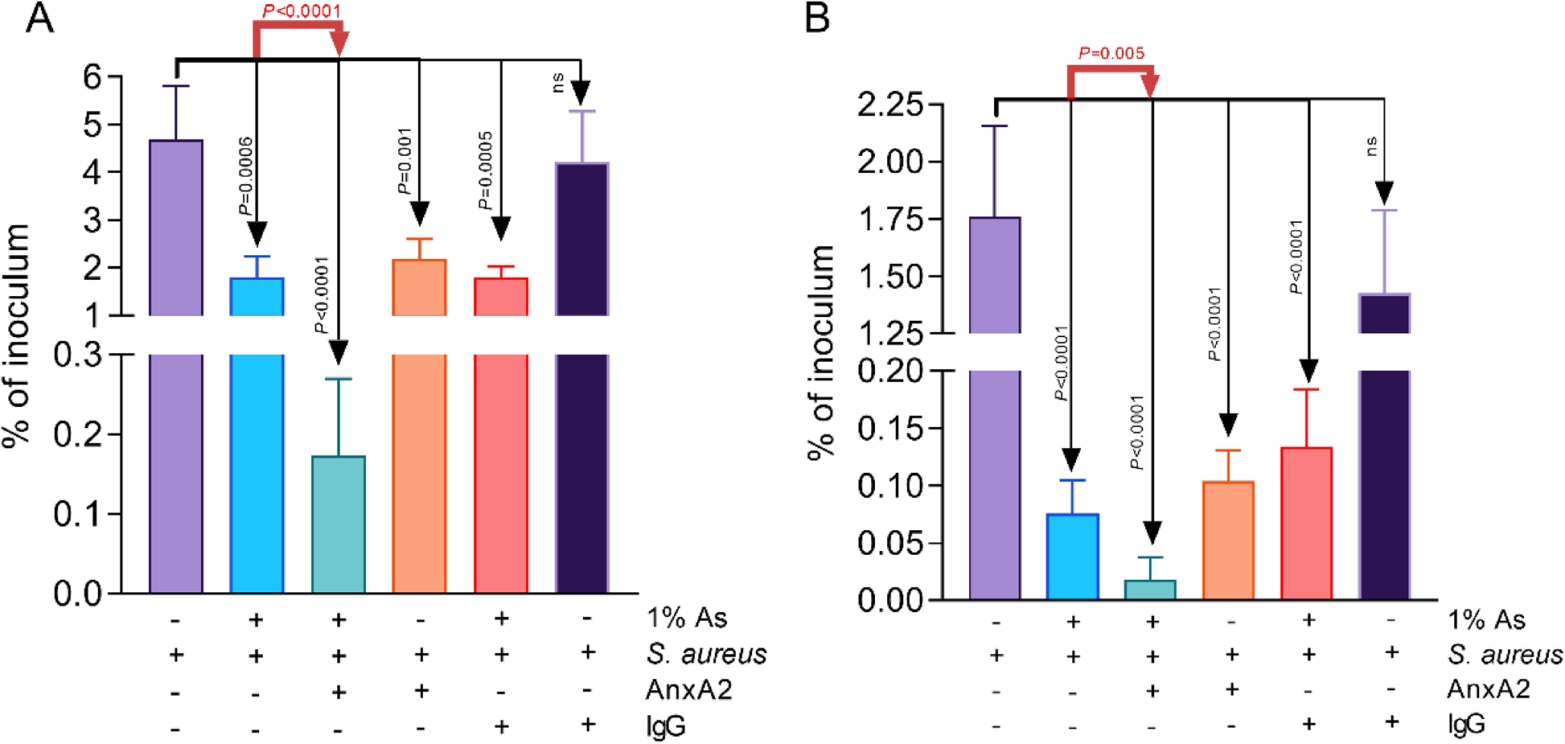
As inactivates AnxA2 and contributes to the reduction in the MAC-T cell invasion of *S. aureus*. **A** adhesion, **B** invasion. All experiments were performed in triplicate and the results are represented as means ± standard deviation (SD). Student’s *t* test was performed to assess significance with the *P* values being indicated on graphs

**Fig. 4:**
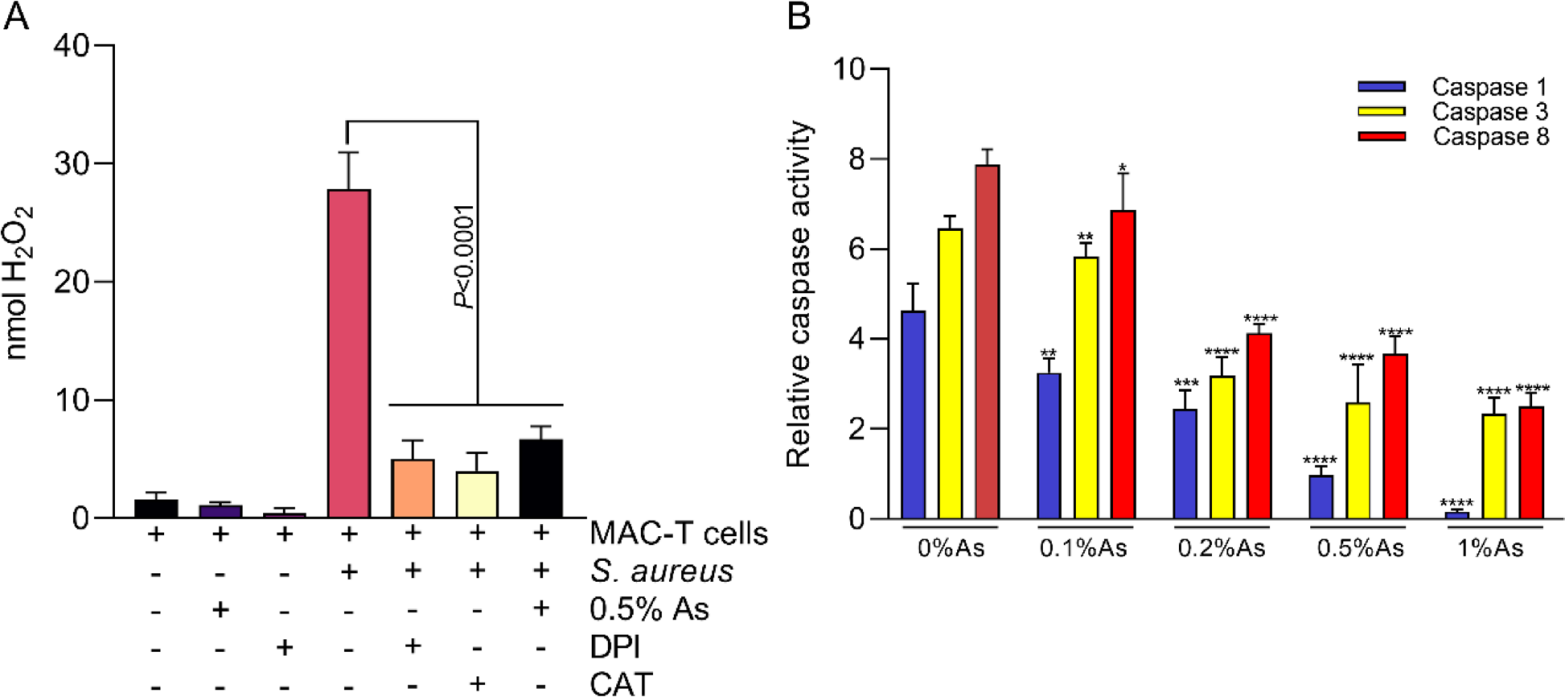
The effect of As on H_2_O_2_ (**A**) and caspases 1,3 and 8 activity (**B**) in *S. aureus* infected MAC-T cells. All experiments were performed in triplicate and the results are represented as means ± SD. A Two-tailed non-parametric *t* test was applied to compare against the 0% As, ****P < 0.0001, ***P = 0.0002, **P ≤ 0.0001, *P < 0.05, *n* = 3

Next, we have investigated if As has a role in the inhibition of cellular Annexin A2 activity, a protein known for interacting with the bacterial ClfB clamping factor (AnxA2) during *S. aureus* adhesion/invasion of MAC-T. To achieve this, we have pre-treated MAC-T cells with AnxA2 antibody for 3 h prior infection with *S. aureus* and the inclusion of As. The total adhesion assay (Fig. 3A) clearly shows that the pre-treatment of MAC-T cells with AnxA2 antibody leads to a further reduction in the total *S. aureus* adhesion (*P* < 0.0001) when compared to As treatment only. Figure 3A also confirms that the adherence levels of *S. aureus* to AnxA2 treated MAC-T cells were similar to the 1% As only experiment, suggesting that they both use a similar adherence-blocking mechanism. As a control, the IgG antibody treatment of MAC-T cells did not affect the bacterial adherence significantly when compared to the un-treated infected control. Similar effects were observed on the numbers of *S. aureus* internalised in the MAC-T cells (Fig. 4B). Taken together, our observations suggest that As blocked the AnxA2 MAC-T cells receptor and prevents its interaction with and combined with the bacterial ClfB receptor resulting decreased infection levels.

### Natural antimicrobial mixture reduces oxidation and caspase activity in S. aureus infected MAC-T cells

Our next aim was to investigate if the reduced bacterial invasion is associated with reduced oxidative and apoptotic stress in the infected MAC-T cells. As shown in Fig. 4A approximatively 28 nmol H_2_O_2_ was released by the infected MAC-T cells in the absence of As. A significant decrease was recorded (*P* < 0.0001) when the infected cells were treated with either 0.5% As, DPI or CAT. These results clearly show that upon infection *S. aureus* triggers the host NADPH oxidases to produce and release H_2_O_2_ by the MAC-T cells, however, this oxidative burst is significantly attenuated in the presence of As. Furthermore, we have investigated if the onset of the oxidative inflammation is also associated with induction of or reduction of caspase 1, 3 and 8 activities in *S. aureus* infected MAC-T cells and in the presence of the antimicrobial mixture. Our results show that all 3 caspases are significantly reduced (Fig. 3B) in a dose dependent manner. Overall, these results show that the natural antimicrobial mixture, As, protects against cellular oxidative damage prevents induction of apoptosis by de-activation of caspase 1, 3 and 8 in the infected MAC-T cells.

## Discussion

The strategy of utilizing natural antimicrobial mixtures fortified with organic acids and botanical extracts offers significant promise for effective pathogen management [21]. Ajose et al. 2023, have recently reviewed ethnoveterinary medicinal plants and ethnoveterinary medicinal products utilised in dairy and livestock farming to combat mastitis, with the most prominent plants as *Becium obovatum*, *Malva parviflora*, *Brucea antidysenterica*, *Acorus calamus* L., *Triticum* sp, *Arachis hypogeam*, *Peganum harmala*, *Citrus limon*, *Withania somnifera* and others [8]. Among plant extracts, Sorghum phenol extract [5], sweet orange peel (*Citrus sinensis*), pomegranate (*Punica granatum*) [22, 23], indian screw tree (*Helicteres isora*) [24], lemon balm (*Melissa officinalis*), a specie of laurel wood (*Knema retusa*) [25] and olive [13] extracts demonstrated strong antimicrobial activity against different *S. aureus* strains including other BM-associated pathogens. After in vitro disc diffusion assays from recent studies, the authors have reported the inhibitory zones against *S. aureus* significant average zones ranging from 22 to 31 mm [24, 26]. The *Knema retusa* extract (KRe) showed significant antibacterial effects at observed MIC between 32 and 256 ug/mL and MBC between 64 and 512 ug/mL against *S. aureus* and *S. haemolyticus*, inhibiting biofilm formation and bacterial surface adherence [25]. In an earlier investigation, a natural antimicrobial mixture of organic acids, citrus and olive extracts was assessed for its anti-staphylococcal activity [13]. This blend manifested a multifaceted impact during the infection process. Initially, it mitigated bacterial virulence attributes like exopolysaccharides (EPS) and biofilm while concurrently compromising bacterial membrane integrity. Further, this mixture hindered the adhesion capability of the *S. aureus* DSM1104 to epithelial cells, bolstered epithelial tight junctions, and did so without compromising cellular viability. Moreover, it notably diminished inflammation and curbed oxidative stress in the infected cells. This antioxidative property can be attributed to its inhibition of externally regulated kinases (ERKs), relevant pathways governing cellular resilience during oxidative challenges and orchestrating the immune response via oxidative dephosphorylation [13].

Natural antimicrobial compounds from EOs could interfere with membrane integrity in bacterial pathogens [27]. Recent findings have showed the potent in vitro antimicrobial and anti-virulent capacities of trans-cinnamaldehyde, thymol, and carvacrol against these challenging pathogens [14]. These compounds reduced the growth of multi-virulent and MDR-MRSA isolates and the transcriptional activity of specific virulence genes. Investigations have further revealed the ubiquitous presence of virulence genes, namely *icaA* and *cna*, among the MRSA isolates studied, with a significant proportion presenting as multi-virulent, potentially complicating their associated infections. Significantly, sub-inhibitory concentrations (SICs) of trans-cinnamaldehyde, thymol, and carvacrol have been found to induce substantial downregulation of genes such as *sea*, *eta*, *tst*, *icaA*, and *cna* in evaluated isolates. Given these insights, the study advocates for the consideration of phytogenic compounds, including trans-cinnamaldehyde, thymol, and carvacrol, as potential alternative antimicrobials to address the growing challenge of MRSA infections [14].

The observed decrease in proinflammatory cytokines, following *S. aureus* infection of MAC-T cells in the presence of various concentrations of Ac, led us to believe that the positive effects might extend to a reduction in other cell survival mediators, such as caspases. For example, Caspase-1 was shown to be involved in the secretion of proinflammatory cytokines, such as interleukin (IL)-1β and IL-18 [28] and known to be involved in apoptosis and inflammation, events which can be inhibited by the natural antimicrobial compounds such as the flavonoids (plant extracts) [29]. These effects were extended to caspases 3 and 8, known for their involvement in apoptosis and the expression of inflammatory cytokines in MAC-T cells with *S. aureus* [20]. The *S. aureus* surface located fibrinogen-binding protein (clumping factor; ClfB) [30] and the cell membrane binding protein Annexin A2 (AnxA2) structurally interact to facilitate bacterial infection of MAC-T cells [31]. Their importance in *S. aureus* invasion of MAC-T cells was further emphasized for the clumping factor A (ClfA) [32]. Our data shows that As is capable of inhibiting ClfB and AnxA2 expression leading to decreased levels of proinflammatory cytokines and reduced bacterial invasion of MAC-T cells, with no need for direct *S aureus* killing. Other natural antimicrobials were also shown to inhibit *S. aureus* biofilm formation through direct binding to ClfB resulting in reduced biofilm formation and cell adhesion [33]. Blocking the functionality of ClfB by natural plant extracts can potentially have a significant impact on the *S. aureus* virulence as it has been previously shown that ClfB can bind to AnxA2 and offer protection against mastitis [31]. Moreover, our study shows that the presence of As during *S. aureus* infection of MAC-T cells resulted in prevention of bacterial adhesion rather than bacterial killing. Previously, it has been shown that AnxA2 can interact with a bacterial receptor, mediating the adhesion to the host cells and ultimately promoting host infection [34]. It was confirmed further that by neutralising AnxA2, *Pseudomonas aeruginosa* infection of mammalian cells was prevented [35]. Similarly, the interaction between ClfB and AnxA2 is vital in *S. aureus* virulence as it has been shown to facilitate bacterial internalisation into MAC-T cells [16].

## Conclusions

In our study we show that As can inhibit AnxA2 activity and bacterial ClfB expression, leading to a significant reduction in the infection levels of *S. aureus* in MAC-T cells. This inhibition mechanism was previously attributed to other natural compounds, like ginseng (G-Rg5 and G-Rk1), which can bind to Annexin A2 resulting in increased apoptosis in cancer cells [36]. We believe that this work will contribute significantly to developing anti-mastitis treatments free of antibiotics and with less impact on the animal or the consumer.

## Data Availability

The datasets used and analysed during the current study are available from the corresponding author on reasonable request.

## Abbreviations

TSAYE: Tryptone Soya Yeast Extract
DMEM: Dulbeccos’ Modifed Eagle medium
MAC-T: Bovine mammary gland epithelial cells
FBS: Foetal bovine serum
MOI: Multiplicity of infection
PBS: Phosphate buffer saline
AnxA2: Annexin A2
